# Methodologic considerations in the design and analysis of nested case-control studies: association between cytokines and postoperative delirium

**DOI:** 10.1186/s12874-017-0359-8

**Published:** 2017-06-06

**Authors:** Long H. Ngo, Sharon K. Inouye, Richard N. Jones, Thomas G. Travison, Towia A. Libermann, Simon T. Dillon, George A. Kuchel, Sarinnapha M. Vasunilashorn, David C. Alsop, Edward R. Marcantonio

**Affiliations:** 1 Division of General Medicine and Primary Care, Beth Israel Deaconess Medical Center, 330 Brookline Ave, CO-203, MA 02215 Boston, Massachusetts USA; 2 Harvard Medical School, Boston, Massachusetts USA; 3 Aging Brain Center, Institute for Aging Research, Hebrew Senior Life, Boston, Massachusetts USA; 4 Division of Gerontology, Beth Israel Deaconess Medical Center, Boston, Massachusetts USA; 5 Department of Psychiatry and Human Behavior, Warren Alpert Medical School, Brown University, Providence, Rhode Island USA; 6 Department of Radiology, Beth Israel Deaconess Medical Center, Boston, Massachusetts USA; 7 Beth Israel Deaconess Medical Center Genomics, Proteomics, Bioinformatics and Systems Biology Center, Division of Interdisciplinary Medicine and Biotechnology, Beth Israel Deaconess Medical Center, Boston, Massachusetts USA; 8 UConn Center on Aging, University of Connecticut Health Center, Farmington, Connecticut USA

**Keywords:** Delirium, Cytokines, Interleukin-6, Case-control, Greedy match, Optimal match, Overmatch, Conditional logistic regression

## Abstract

**Background:**

The nested case-control study (NCC) design within a prospective cohort study is used when outcome data are available for all subjects, but the exposure of interest has not been collected, and is difficult or prohibitively expensive to obtain for all subjects. A NCC analysis with good matching procedures yields estimates that are as efficient and unbiased as estimates from the full cohort study. We present methodological considerations in a matched NCC design and analysis, which include the choice of match algorithms, analysis methods to evaluate the association of exposures of interest with outcomes, and consideration of overmatching.

**Methods:**

Matched, NCC design within a longitudinal observational prospective cohort study in the setting of two academic hospitals. Study participants are patients aged over 70 years who underwent scheduled major non-cardiac surgery. The primary outcome was postoperative delirium from in-hospital interviews and medical record review. The main exposure was IL-6 concentration (pg/ml) from blood sampled at three time points before delirium occurred. We used nonparametric signed ranked test to test for the median of the paired differences. We used conditional logistic regression to model the risk of IL-6 on delirium incidence. Simulation was used to generate a sample of cohort data on which unconditional multivariable logistic regression was used, and the results were compared to those of the conditional logistic regression. Partial R-square was used to assess the level of overmatching.

**Results:**

We found that the optimal match algorithm yielded more matched pairs than the greedy algorithm. The choice of analytic strategy—whether to consider measured cytokine levels as the predictor or outcome-- yielded inferences that have different clinical interpretations but similar levels of statistical significance. Estimation results from NCC design using conditional logistic regression, and from simulated cohort design using unconditional logistic regression, were similar. We found minimal evidence for overmatching.

**Conclusions:**

Using a matched NCC approach introduces methodological challenges into the study design and data analysis. Nonetheless, with careful selection of the match algorithm, match factors, and analysis methods, this design is cost effective and, for our study, yields estimates that are similar to those from a prospective cohort study design.

## Background

Nested case-control study (NCC) design within a prospective cohort study is used when the outcome data are available for all subjects, but the exposure of interest has not been collected, and is difficult or prohibitively expensive to obtain for all subjects [1–3]. NCC is cost effective and can be done with or without matching in the selection of a subset of the controls. NCC analysis with good matching procedure yields estimates that are as efficient and unbiased as estimates from the full cohort study [2]. The origin of the NCC design came from the desire to reduce computational costs of collecting and analyzing data for all subjects in a cohort study. Mantel proposed to sample the controls randomly from a finite cohort, and originally called this design “synthetic” case-control study [4]. Subsequently the use of matching to select the controls allowed for the implementation of the conditional likelihood functions and the demonstration of asymptotic consistency and efficiency property of the risk ratio estimates [5]. NCC has been used in many biomarker studies where it is expensive to collect and process biological samples for all subjects in the cohort study. Recent applications of NCC include studies showing the effects of serum lipids and lipoproteins on breast cancer risk [6], urine semaphorin-3A on renal damage in hypertensive patients [7], DNA methylation markers on type-2 diabetes [8], and plasma cytokines and the risk of HIV type one [9].

In addition to being cost effective, NCC with a smaller sample size tends to be less computationally demanding than the analysis of the full cohort study. If the match procedure is carried out properly, and the selected controls are representative of the controls in the cohort study, then NCC loses little efficiency compared to the full cohort analysis [3]. NCC could offer better validity than the full cohort study because the match procedure allows for adjustment for both measured, and for unmeasured confounders [10].

At the crux of the NCC design is the quality of the match procedure and the appropriate analysis that accounts for the match design. Algorithms used in match procedures such as the greedy algorithm, propensity score algorithm, and optimal algorithm are some of the most often used in NCC studies. Theoretically, the optimal algorithm has been demonstrated to outperform the greedy algorithm at the expense of computational costs [11]. However, in the context of our clinical study where the number of needed matched pairs was smaller than 50, it was not clear how large the difference would be in the performance of these algorithms. In terms of the interpretation of the analysis results, in the case of a binary outcome and a binary exposure, one can compute the odds ratio for the outcome, or the odds ratio for the exposure, and these two odds ratio estimators have been shown to be equivalent [12]. However, in the case of a binary outcome, and a continuous exposure (such as in biomarker discovery studies where the exposure is the level of putative marker concentration in plasma), it is not clear that the outcome odds ratio is equivalent to the mean or median of the paired differences (between the case and control in a selected pair). For a NCC with matching, Cornfield [12], Mantel and Haenszel [13], Breslow [2], Rubin [14], Rothman and Greenland [15] have pointed out that the match algorithm, the match factors, and their association with the outcome and the exposure play a critical role in validity and efficiency. In addition, caution is needed to avoid overmatching, since this could introduce bias and inefficiency into the estimators.

As a case study to evaluate these issues, we used a clinical study that focused on estimating the association between cytokines and postoperative delirium [16–19], a common and serious clinical syndrome that is associated with a sudden decline in attention and cognition. The study used a large cohort of older adults undergoing non-cardiac surgery enrolled in the SAGES: Successful Aging after Elective Surgery study [20, 21]. Because we planned to employ high cost, labor intensive biomarker discovery technologies such as Luminex multiplex cytokine panels and proteomics using mass spectrometry, it was realistic to only measure the biomarkers in a subset of the patients. Therefore, we chose a NCC design. Cases with delirium were matched to a subset of controls without delirium. Controls were chosen based on the match of six demographic and baseline clinical variables thought to be potential confounders of the cytokine/delirium association.

To address the methodological issues described above, we set out to answer the following questions in this paper: 1) How much better, in terms of the number of selected matched pairs, and the quality of the match in a selected pair, is the optimal match algorithm compared to the greedy algorithm, and the propensity score algorithm? 2) Should we treat postoperative delirium incidence as the outcome and report the odds ratio estimates from the conditional logistic regression models, or should we treat cytokine (specifically, IL-6) concentration as the outcome and report the median paired difference between the delirium cases and controls? 3) Compared to the full cohort analysis, in our case with simulated IL-6 for the full cohort, how efficient and valid would the estimates from the NCC study be (both odds ratios from the conditional logistic regression model, and the median paired difference analysis)? 4) Is there evidence for overmatching, and how should that be quantified and interpreted?

## Methods

### Description of study data

The SAGES study has been described in detail previously [20, 21]. Briefly, the study enrolled 566 adults age ≥70 who were scheduled for major non-cardiac surgery. Demographics and baseline clinical information such as comorbid conditions and cognitive function were collected preoperatively. During hospitalization, patient’s delirium status was assessed during daily interviews using the Confusion Assessment Method (CAM) and chart review. Functional and cognitive data and other outcomes were collected at baseline and at each of the follow-up time point. Blood samples for each patient were collected prior to surgery (PREOP), in the post anesthesia care unit (PACU), 2 days after surgery (POD2). One of the aims of the project was the estimation of the association between cytokines and postoperative delirium. After testing several kits in our pilot work, we chose the Luminex high-sensitivity kit from R&D Systems Inc. with 12 inflammatory cytokines to obtain estimated concentrations (pg/ml) at each of these 3 time points. To evaluate the methodology used in our NCC design for this paper, we will use only IL-6 as a representative cytokine.

### Definition of delirium case and control

A case was defined as having [delirium on POD2], or [delirium on POD1 and subsyndromal (partial) delirium on either POD2 or POD3]. This definition was used to ensure that the blood sample on POD2 was reflective of the delirious state. A control was defined as not having delirium or subsyndromal delirium on any hospital day. We also required cases and controls to have blood samples with no or only mild hemolysis (a condition that may contaminate the plasma and cause inaccurate laboratory measurements). Our recent published work has more details on this issue [22]. At the time of this analysis, there were 272 subjects, 49 met the case definition, and 143 met the control definition. From these, 39 matched pairs were selected.

### Match factors in the case-control design

Six factors were judged to be potentially confounding the association between cytokines and delirium. These factors were largely selected based on prior literature, clinical experience of the investigators and the Program Project Operations Committee that oversees the study. The six factors were: 1) age at surgery, 2) gender, 3) vascular comorbidity (having any one of the six conditions: myocardial infarction, congestive heart failure, peripheral vascular disease, cerebrovascular disease, diabetes, and diabetes with end organ damage), 4) surgery type (orthopedic, vascular, gastrointestinal), 5) presence of the ApoE ɛ4 allele, 6) baseline GCP (general cognitive performance score, a summary measure derived from a detailed neurocognitive battery [23]. Among the match factors, only age and GCP were continuous variables. The other 4 match variables were categorical. After discussion with the study team, we decided to create our match algorithms such that we required an identical value between the case and the control on the four categorical variables, and a difference (caliper) of no more than five years for age, and no more than five points for GCP. Table 1 shows the distribution of the six match factors before and after the match. Of the 49 eligible cases and 143 eligible controls, 39 match pairs were created. The match cohort has identical prevalence for case and control on four categorical match variables (gender, surgery type, vascular comorbidity, APOE ɛ4), and similar mean and standard deviation for age and baseline GCP (Table 1).

**Table 1:** Distribution of Demographic and Baseline Clinical Characteristics

	Number of Subjects	Age at Surgery (M ± SD)	Baseline GCP (M ± SD)	Gender (% Female)	Surgery Type (% Orthopedic)	Vascular Comorbidity (%)	APOE ε4 carrier (%)
Pre-match Delirium Case	49	77.2 ± 4.9	54.2 ± 5.9	55%	86	45	18
Pre-match Control	143	76.3 ± 4.8	58.9 ± 6.6	57%	85	29	24
Post-match Delirium Case	39	77.3 ± 5.1	55.2 ± 5.6	54%	92	38	13
Post-match Control	39	76.8 ± 4.7	56.4 ± 5.2	54%	92	38	13

*M* mean, *SD* standard deviation, *GCP* general cognitive performance, *APOE* Apolipoprotein E

### Issue 1. Performance of match algorithms

We first considered three candidate match algorithms: propensity score [24], greedy [25], and optimal [11]. We eliminated the propensity score approach because the match was required to be exact for 4 out of 6 factors, and the propensity score method cannot guarantee this outcome. We then evaluated the two remaining algorithms: the greedy match, and the optimal match algorithm. The greedy match algorithm is widely used and implemented in many statistical procedures for matching and is computationally faster than the optimal match. However, in terms of the match quality, that is, the degree of similarity (measured by absolute, Euclidean, or Mahalanobis distance on the match factors between the case and the control), the optimal match algorithm has been shown to outperform the greedy algorithm [11]. Also, the optimal algorithm theoretically will yield a greater number of matches than the greedy algorithm. We will now briefly illustrate the application of both the greedy and optimal algorithms in our study.

For each case, the greedy algorithm first evaluates each of the controls and measures the total distance (we used the absolute value) from the six match factors between the case and the control. The requirement for a match is to have identical values on four categorical variables (required distance of zero), and no more than a 5-unit difference on each of two continuous variables. Thus for a match to be successful, the total distance must be less than ten units. The best match would have a total distance of zero. The larger the distance on the continuous variables, the worse the quality of the match.

The greedy algorithm evaluates all the controls that meet this requirement, and selects the control that has the minimum distance to a case to form a match pair. Both the case and control for this match pair are then eliminated from the pool of match eligible cases and controls. The optimal match algorithm is similar to the greedy algorithm; however, once the match pair is formed, its case and control are not eliminated from the pool, but rather can be uncoupled and matched again if the total distance up to that point with a new control or a new case is smaller. For a large dataset, it is this reconsideration to attain minimum total distance that makes the optimal match algorithm more computationally consuming than the greedy match.

Given the small sample size, we also performed the match algorithm based on at 1:2 design (one case for two controls). We aimed to assess if the results in the 1:1 NCC would hold when the sample size gets larger.

### Issue 2. Choice of exposure vs. outcome

In analyzing data from this study, we were faced with a choice of treating the IL-6 levels as the predictor and delirium as the outcome versus making delirium the predictor and IL-6 the outcome. The former would involve reporting the odds ratio of delirium per unit increase (pg/ml) of IL-6 by using conditional logistic regression at the 3 time points [26, 27], while the latter would result in reporting the mean or median of the paired matched case-control differences in IL-6 levels. In our case, IL-6 distributions were not normally distributed and the nonparametric approach [28] was more appropriate; therefore, the median paired difference (MPD) would be used to test for the null hypothesis of MPD equal to zero using the signed rank test.

Earlier work by Cornfield [12], showed that in the case-control design, if the exposure is binary, then the exposure odds ratio is indeed equal to the disease odds ratio. This is also true for the NCC design. In other words, treating delirium as either the outcome or exposure in the analysis would yield identical odds ratio estimate. In our case with the exposure variable IL-6 being continuous, it is no longer true that the estimated MPD can be equated to the disease (delirium) odds ratio. The two estimates also carry different clinical interpretations. The MPD conveys a longitudinal change of IL-6 through time due to delirium; whereas, the odds ratios from conditional logistic models help to assess how IL-6 influences delirium risk across different time points. After considering both approaches, we elected to use the MPD in the paper [22]. We felt it would be more informative to show how the median levels of cytokines varied over time in both the delirium and non-delirium groups. Here in this paper, we present both analysis methods, and examine the differences in the two estimators and their interpretations.

### Issue 3. Nested case-control versus cohort design and analysis

A second analytic issue was our interest in comparing the NCC results to those of the cohort study results assuming that the data for the cohort study design were available. Mantel and Haenszel [13], and Breslow [2] articulated that the data from case-control study design could be thought of as a random sample, based on the outcome data, from the prospective cohort design; therefore, one should expect the case-control design to yield similar estimates as the cohort design. The difference in the estimators between the case-control and cohort design could indicate bias, which could be attributed to sources such as bias selection of the sampled controls, and/or the match factors, and the match algorithm in the case-control design. We hypothesized that the NCC match analysis using conditional logistic regression would yield estimates which are similar to those of the simulated cohort analysis and the conclusion about the IL-6 effect would be the same in both analysis methods.

To address this issue, since we only measured IL-6 in the 39 pairs of cases and controls, we used simulation to generate the IL-6 data for the whole cohort of study participants who were eligible for the match (*N* = 192). In addition to the 114 subjects who did not get matched, we decided to simulate IL-6 data also for the 78 subjects in the NCC study so that all subjects would have the same probability of being assigned randomly an IL-6 measurement. Based on the time point, and delirium status of the subject, a randomly selected measured IL-6 from the 78 matched subjects with the corresponding time point and delirium status was assigned, with replacement. We ran the simulation once and created one simulated dataset because the pool of measured IL-6 values (*N* = 78) to sample from was smaller than the number of required simulated values (*N* = 192). Using sampling with replacement, repeated samples would have been quite similar, and therefore one simulated sample was felt to be sufficient. The cohort analysis using simulated IL-6 data used a multivariable unconditional logistic regression with the independent variables being IL-6 and the six match factors, and the dependent variable being the binary delirium outcome variable.

### Issue 4. Assessment of overmatching

A known phenomenon of the match algorithm in the NCC design is called overmatching, which can introduce bias and inefficiency into the estimation of the case-control study. Breslow [1] reported an in-depth simulation for a number of scenarios using a single binary match factor, one binary outcome, and one binary exposure. When there is no association between the match factor and exposure, or when there is no association between the match factor and the outcome, there is no need to use matching and stratified analysis (such as conditional logistic regression). One can just use a random sample of the controls, and use a non-stratified analysis. In fact, if matching is used, then the estimated odds ratio would be biased toward the null, and the variance of the odds ratio estimate would be inefficient (that is larger than that of an estimator derived from a random sample of the controls). Another situation is when there is an association between the match factor and the exposure, and an association between the match factor and the outcome. Matching would select controls with exposure value similar to that of the cases, leading to bias toward the null. The magnitude of this bias increases as the association between the match factor and the exposure increases. If matching is not used in this case, but a random sample of the controls is selected, then the bias is in fact worse, and goes away from the null. Thus, in this situation, no analysis solution would be available to fix the bias issue [1, 2].

For our study, it was impossible to assess the association between the exposure IL-6 and the outcome delirium, with the six match factors at the design phase of the NCC study, because we did not have IL-6 data. We selected the six match factors using strictly clinical experts’ input and review of the literature. After the NCC study design had been implemented, and IL-6 was measured, we now know that there is an association between the exposure (IL-6) and the outcome (delirium incidence) on POD2. We also know that the match factors are jointly associated with the outcome. Thus the degree of bias in the estimate of the association between IL-6 and delirium depends on the association between IL-6 and the match factors. That is, if the joint distribution of the six match factors is associated with IL-6 concentration, then the estimation of IL-6 effect on postoperative delirium in the conditional logistic regression model could be underestimated (biased toward the null).

The reason for this phenomenon can be illustrated with an example. Assume that one of the match factors, vascular comorbidity, is associated with IL-6. So those subjects with the presence of vascular comorbidity have higher level of IL-6 than those who do not have this comorbidity. Take a matched pair of a case and a control subject who both had vascular comorbidity. The values of IL-6 for this pair would both be high because vascular comorbidity is associated with IL-6. Take another pair who did not have vascular comorbidity. This pair would have both IL-6 values in the lower range relative to the pair with vascular comorbidity. Thus, the difference of IL-6 between members of the pair, for each of these two pairs, would be small, and yield small IL-6 difference. If vascular comorbidity was not associated with IL-6, then within a pair, the value of IL-6 could be high for one member and low for the other, leading to a larger difference, and thus a stronger effect of IL-6. This is the effect of overmatching.

To assess overmatching, we used only the data of the controls. Overmatching was possibly due to not a single match factor, but rather a joint distribution of 6 factors that would be required to be associated with the biomarkers to potentially cause the underestimation of IL-6 effect. To further evaluate whether this was the case, we used a general linear model where the dependent variable was IL-6 concentration, and the independent variables were the six match factors. The strength of the association between the joint distribution of the match factors and IL-6 was measured by the R-squared estimate. From this linear model, using partial R-squared estimates, we also decomposed the model R-squared into individual components which reflect the association of each match factor on IL-6.

All data management and analyses for this paper were carried out using the SAS software. For the simulation, we used procedure SURVEYSELECT in SAS/STAT software [29] with the method of unrestricted random sampling (URS) which allows selection of subjects with equal probability and with replacement.

## Results

**Fig. 1:**
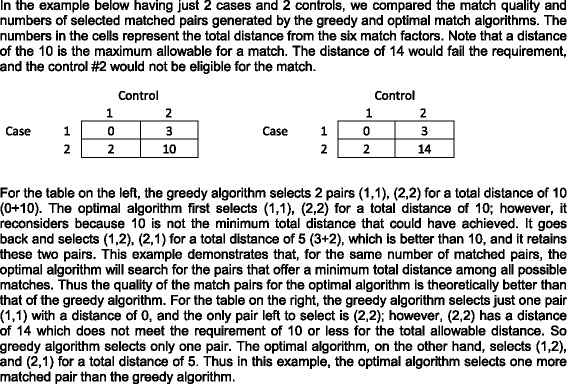
Illustration of the difference between greedy and optimal match algorithm. A numerical example is given here to demonstrate the theoretical properties of the greedy and optimal match algorithm

For issue 1, evaluating the performance of the match algorithms, we illustrated in Fig. 1, with just 2 cases and 2 controls, a theoretical exercise demonstrating how both algorithms select the controls, and how the optimal algorithm yielded more match pairs with better quality than the greedy algorithm. To further illustrate the property of the greedy vs. optimal match algorithms using our data, in Table 2, we conducted an exercise where we varied the caliper of age (the difference of age in years between the case and control in the match pair) and GCP (the difference in GCP units between the case and control in the match pair) from one to five units to compare the performance of the greedy and optimal match algorithms. The optimal algorithm yielded more matched pairs than the greedy algorithm at caliper four (34 pairs vs 32 pairs), and at caliper five (39 pairs vs 34 pairs). If the same number of pairs was chosen by both algorithms, the optimal algorithm yielded higher quality pairs (with smaller mean distance, for example 2.05 versus 2.06 for caliper of 3). Note that we used a fixed caliper of five for the actual study, and did not vary it as we did in this exercise.

**Table 2:** Performance of Greedy and Optimal Algorithm at Different Levels of Caliper

Match Algorithm	Age Caliper	GCP Caliper	Number of Pairs	Mean Distance	Sum of Distance
Greedy	1	1	7	0.99	6.90
Optimal	1	1	7	0.99	6.90
Greedy	2	2	21	1.79	37.51
Optimal	2	2	21	1.76	36.89
Greedy	3	3	26	2.06	53.63
Optimal	3	3	26	2.05	53.48
Greedy	4	4	32	2.68	85.81
Optimal	4	4	34	2.99	101.7
Greedy	5*	5	34	3.01	102.45
Optimal	5*	5	39	3.79	147.79

*Caliper is defined to be the minimum allowable difference between the case and the control. In our study, we used a caliper of five for age and GCP, and zero for the other four categorical variables

*GCP* general cognitive performance

We also performed a 1:2 design to see if the observation we saw in the 1:1 design holds. Table 3 below shows the result for both the optimal, and greedy match algorithm. Each case is set to match to two controls, but this was not always possible. So when there were not two controls available, one control was chosen. As a result, we have some cases with two controls and some cases with only one control. For example, for caliper one for age, and GCP, a total of eight pairs was obtained from seven cases. Six of seven cases matched to one controls, but there was one case that matched to two controls. Note that when the caliper of age and GCP was tight, for example, as one or two or even three, it was harder to get a match, and therefore, there were more 1:1 match pairs (one case to one control) than 1:2 match pairs. As the caliper got wider as in five and six, it was easier to satisfy the match criteria, and so there were more 1:2 matches than 1:1 (e.g. at caliper five, for the optimal match, there were 24 1:2 matched pairs and 12 1:1 match pairs; and at caliper six, there were 28 1:2 matches versus 11 1:1 matches). Notice that beginning at caliper four, the optimal algorithm yielded more match pairs than the greedy algorithm; therefore, even with 1:2 design, our conclusion on the superiority of the optimal algorithm holds true, as in the case of 1:1 match design (Table 2). Of note, when we expanded the caliper width to six units for age and GCP, as in Table 3 shows, the result still holds, the optimal algorithm yielded five more match pairs than the greedy algorithm. On the basis of its demonstrated superiority in both quality and quantity of matches, we chose the optimal algorithm for our matched, NCC study design.

**Table 3:** Performance of Greedy and Optimal Algorithm at Different Levels of Caliper for a 1:2 Match

Match Algorithm	Age Caliper	GCP Caliper	Number of Pairs	Number Cases/Controls^b^	Mean Distance	Sum of Distance
Greedy	1	1	8	7/6/1	1.02	8.15
Optimal	1	1	8	7/6/1	1.02	8.15
Greedy	2	2	28	21/14/7	1.90	53.29
Optimal	2	2	28	21/14/7	1.90	53.29
Greedy	3	3	38	26/14/12	2.28	86.54
Optimal	3	3	38	26/14/12	2.28	86.63
Greedy	4	4	48	32/16/16	2.87	137.9
Optimal	4	4	49	33/17/16	2.97	145.7
Greedy	5^a^	5	56	34/12/22	3.47	194.4
Optimal	5^a^	5	60	36/12/24	3.91	234.4
Greedy	6	6	62	37/12/25	3.98	247.1
Optimal	6	6	67	39/11/28	4.27	286.1

^a^Caliper is defined to be the minimum allowable difference between the case and the control. In our study, we used a caliper of five for age and GCP, and zero for the other four categorical variables

*GCP* general cognitive performance

^b^There are three numbers listed in this column, for example 7/6/1 means seven cases were matched, six cases matched to 1:1, so six controls; and one case matched to 1:2, so two controls. This gives a total of eight matched pairs

For issue 2, evaluating the choice of exposure versus outcome, we analyzed the data using conditional logistic regression for the observed IL-6, and nonparametrically using the signed rank test [28] on the median of paired differences (Table 4). Inferentially, from both methods, the differences between cases and controls were not statistically significant at PREOP, PACU, but were significant at POD2 for both methods (*p* = 0.005). The odds ratio estimate for POD2 was 1.02 (95% CI: 1.01–1.03), and the MPD was 50.44 pg/ml. While the level of statistical significance was the same, the two estimates convey different interpretations: one expressed a 2% increase in the odds of delirium incidence per one pg/ml increase in IL-6, and the other yielded an estimate of the population median difference of IL-6 between delirium cases and controls on POD2. Ultimately, it is reassuring that the two analytic approaches yielded the same conclusions in terms of a statistically significant association between IL-6 and delirium at POD2, despite yielding different effect measures with different clinical interpretations.

**Table 4:** Comparison of Analytic Approaches Using Data on Delirium and Interleukin-6 (IL-6)

	Number of Subjects	PREOP	PACU	POD2
(a) Matched Analysis of Delirium as the Outcome vs IL-6 as the Outcome				
Matched Analysis^a^				
Delirium as the Outcome	78 (39:39)			
Beta (SE)		0.00745 (0.0112)	0.00321 (0.00327)	0.0154 (0.0054)
*P*-value		0.508	0.326	0.005
Odds Ratio (95% CI)		1.01 (0.99–1.03)	1.00 (0.99–1.01)	1.02 (1.01–1.03)
Matched Analysis^b^				
IL-6 as the Outcome	78 (39:39)			
MPD		1.11	9.13	50.44
*P*-value		0.475	0.123	0.005
(b) Matched Analysis Using Observed IL-6 vs. Unmatched Analysis Using Simulated IL-6				
Unmatched Analysis				
Simulated IL-6^c^	192 (49:143)			
Beta (SE)		-0.0385 (0.0322)	0.00323 (0.002)	0.0176 (0.004)
*P*-value		0.231	0.105	0.0001
Odds Ratio (95% CI)		0.96 (0.90–1.03)	1.00 (0.99–1.01)	1.02 (1.01–1.03)
Matched Analysis				
Observed IL-6^d^	78 (39:39)			
Beta (SE)		0.00745 (0.0112)	0.00321 (0.00327)	0.0154 (0.0054)
*P*-value		0.508	0.326	0.005
Odds Ratio (95% CI)		1.01 (0.99–1.03)	1.00 (0.99–1.01)	1.02 (1.01–1.03)

^a^The coefficients (Beta and SE), *p*-values, and odds ratios came from the conditional logistic regression where delirium case was the outcome, and IL-6 the independent variable

^b^The MPD (median paired differences) is the median of the paired differences (concentration level of IL-6 from the delirium case minus that of the control) within each time period. The *p*-values came from the nonparametric signed rank test

*PREOP* preoperative, *PACU* post-anesthesia care unit, *POD2* postoperative day 2, *SE* standard error, *CI* confidence interval

^c^The coefficients (Beta, SE), *p*-values, and odds ratios came from the unconditional multivariable logistic regression with delirium case as the outcome, and the six match factors as independent variables

^d^The coefficients (Beta, SE), *p*-values, and odds ratios came from the conditional logistic regression where delirium case is the dependent variable, and IL-6 the independent variable

*PREOP* preoperative, *PACU* post-anesthesia care unit, *POD2* postoperative day 2, *SE* standard error, *CI* confidence interval

We also carried out a sensitivity analysis assessing the influence of IL-6 values in the right hand tail of the distribution. We focused on POD2 because this was the time period that we found significant IL-6 effect on postoperative delirium (Table 4, the matched analysis with conditional logistic regression). The question here is if the inference would change if we did not include potentially influential large IL-6 values (e.g. above 90^th^, 95^th^ percentile of the IL-6 distribution) in the modeling. At the other two periods, PREOP and PACU, the findings were highly non-significant. The distribution of IL-6 on POD2 has a mean of 109.7, SD = 81.2, median = 93.7, minimum = 3.98, maximum = 410.5. As shown in Table 4, including all IL-6 data yielded the log OR estimate of 0.0154 (SE = 0.0054) and *p*-value = 0.005. When we only included data below the 90^th^ percentile of the distribution (cut off IL-6 at 192), the log OR estimate = 0.0150 (SE = 0.0061) and *p*-value = 0.0135. When we included data below the 95^th^ percentile (cut off IL-6 at 316), the log OR estimate = 0.0148 (SE = 0.0056) and *p*-value = 0.0087. So these estimates changed slightly and the significant effect of IL-6 remains.

For issue 3, evaluating the two types of analyses: nested case-control, and cohort design, we compared, in Table 4, the results obtained from the NCC design vs. the simulated cohort design. The NCC study and the unmatched cohort analysis yielded the same conclusion: no significant effect of IL-6 on PREOP, PACU, and significant effect on POD2. The point estimates of the odds ratios, and 95% confidence intervals were almost identical for POD2 between the two analysis methods (odds ratio = 1.02, 1.01–1.03). Out of the three time points, one has lower standard errors in the match analysis (PREOP). From this simulated analysis, we concluded that our match NCC design with *N* = 78 yielded nearly identical point estimates and had similar statistical efficiency as a more traditional cohort design with *N* = 192.

For issue 4, from Table 5, we found that the largest R-squared estimate was 22.30% from the PREOP period, which is equivalent to a correlation coefficient of 0.47. In the PACU too, gender has the largest partial R-squared estimate of 8.38%, which is about 0.29 for correlation. No variables exceeded 9% for partial R-squared estimation. Due to the relatively low magnitude of these individual factor partial R-squared estimates, we believe that the bias due to overmatching is not a major issue in our design and analysis.

**Table 5:** Association between Match Factors and interleukin-6 (IL-6) from Controls

	PREOP (%)	PACU (%)	POD2 (%)
Combined Match Factor R-squared	22.3	18.5	21.8
Individual Match Factor			
Partial R-squared			
Age at Surgery	3.69	7.63	0.27
Baseline GCP	4.12	1.17	1.34
Gender	8.62	8.38	7.65
Surgery Type	0.37	0.37	1.96
Vascular Comorbidity	1.00	0.28	8.76
APOE	4.45	0.62	1.84

R-squared estimated from the general linear model where IL-6 was the dependent variable, and the six match factors were the independent variables. None of the variables were statistically significant in the linear models except Gender in the PREOP period

*GCP* general cognitive performance, *APOE* apolipoprotein E

## Discussion

In this paper, we discuss methodological issues related to the nested, matched case-control design, which is being increasingly used in biomarker discovery studies. We discussed the potential advantages of such a design, as well as the resultant complexities in analysis and interpretation of the results.

By using the NCC study design rather than a more traditional cohort design, we performed biomarker assays on only a portion of the cohort, resulting in a substantial cost savings. The tradeoff in the case-control design was the potential loss of efficiency and presence of bias in our estimation. These two potential drawbacks could come from the match algorithm employed, the approach used for modeling of the data, and from overmatching. We evaluated two candidate match algorithms and found that the optimal algorithm was superior to the greedy algorithm in yielding more match pairs with higher match quality. The interesting lesson that we learned is that with a match design of 1:2 or 1:3, we could increase the match pairs and thus statistical power to the analysis; however, the analysis could become more complicated due to the lack of independence among the match pairs. This dependency would have made our MPD analysis which uses nonparametric signed rank test invalid since the signed rank test requires independence.

Our evaluation of the IL-6 association with postoperative delirium needs careful clinical interpretation. We hypothesized that if the association between PREOP IL-6 and delirium case was statistically significant at type-I error of 0.05 then we would consider PREOP IL-6 a risk marker. This definition also applied to PACU IL-6.

Another issue we encountered was whether to use an analytic strategy in which delirium was the outcome or the predictor. We compared the strategies of using non-parametric signed rank test (delirium is the predictor and median IL-6 levels are the outcome) vs conditional logistic regression (IL-6 levels are the predictor and delirium is the outcome). We found similar results in terms of statistical significance, although we felt the former approach yielded more clinically meaningful effect estimates. We used simulation to evaluate the impact of whether a cohort study would have yielded similar results to our NCC study. Using unmatched multivariable logistic regression modeling, we found the results of this simulated cohort study to be very similar to those of the chosen NCC study design. Finally, to assess for overmatching, we also checked for the representativeness of our match sample in comparison to the pre-matched sample, and found that in both the controls, and cases, the post-matched sample of 78 subjects was quite similar to the pre-match sample of 193 subjects. In addition, we conducted a post-hoc evaluation of the correlation of the measured IL-6 levels with the joint effect of our six match variables in the sample of post-matched controls and found low partial r-square estimates for the individual match factor in each time period. This finding allows us to conclude that the likelihood of bias toward the null due to overmatching was not a major issue in the analysis. We also checked for the similarity in distribution of the match factors between the 39 controls after the match, and the 143 controls before the match and found the two samples to be similar in five of the six variables. Given the small sample size that we have, the statistical tests to compare the distributions between pre-match and post-match controls, and cases may not have sufficient power to detect statistical significance. Clinically, we think that of the six factors, only APOE e4 may show a clinically relevant difference between the two samples.

We also examined the overmatching issue and found the partial r-square estimates of the six match factors to be small. These analyses indicate that selection bias in the controls is a minor issue.

## Conclusions

Past studies in the literature indicated that NCC design has been used widely; however, most focused on the application of the design. For delirium research, in particular in the area of biomarker study, we are not aware of any published study which explores the methodological issues of the NCC design. Therefore, this detailed assessment of the methodological issues in our NCC study design provides insights that will inform the design of future studies, particularly in the field of biomarker discovery.

## Abbreviations

ApoE: Apolipoprotein E gene
CAM: Confusion assessment method
DNA: Deoxyribonucleic acid
GCP: General cognitive performance
IL-6: Interleukin-6
MPD: Median paired difference
NCC: Nest case-control study
PACU: Post anesthesia care unit
pg/ml: Picogram per milliliter
PO1MO: Postoperative at 1 month
POD2: Postoperative day 2
POD3: Postoperative day 3
PREOP: Preoperative
SAGES: Successful aging after elective surgery
